# The effect of smoking on latent hazard classes of metabolic syndrome using latent class causal analysis method in the Iranian population

**DOI:** 10.1186/s12889-023-16863-6

**Published:** 2023-10-20

**Authors:** Farzad Khodamoradi, Maryam Nazemipour, Nasrin Mansournia, Kamran Yazdani, Davood khalili, Maedeh Arshadi, Mahyar Etminan, Mohammad Ali Mansournia

**Affiliations:** 1 Department of Social Medicine, Faculty of Medicine, Ahvaz Jundishapur University of Medical Sciences, Ahvaz, Iran; 2 Department of Epidemiology and Biostatistics, School of Public Health, Tehran University of Medical Sciences, PO Box: 14155-6446, Tehran, Iran; 3 Department of Endocrinology, AJA University of Medical Sciences, Tehran, Iran; 4 Prevention of Metabolic Disorders Research Center, Research Institute for Endocrine Sciences, Shahid Beheshti University of Medical Sciences, Tehran, Iran; 5 Department of Epidemiology and Biostatistics, School of Public Health, Shahid Sadoughi University of Medical Sciences, Yazd, Iran; 6 Departments of Ophthalmology and Visual Sciences, Medicine and Pharmacology, University of British Columbia, Vancouver, Canada

**Keywords:** Cohort studies, Smoking, Metabolic syndrome, Causal analysis, Latent class analysis, Propensity score

## Abstract

**Background:**

The prevalence of metabolic syndrome is increasing worldwide. Clinical guidelines consider metabolic syndrome as an all or none medical condition. One proposed method for classifying metabolic syndrome is latent class analysis (LCA). One approach to causal inference in LCA is using propensity score (PS) methods. The aim of this study was to investigate the causal effect of smoking on latent hazard classes of metabolic syndrome using the method of latent class causal analysis.

**Methods:**

In this study, we used data from the Tehran Lipid and Glucose Cohort Study (TLGS). 4857 participants aged over 20 years with complete information on exposure (smoking) and confounders in the third phase (2005–2008) were included. Metabolic syndrome was evaluated as outcome and latent variable in LCA in the data of the fifth phase (2014–2015). The step-by-step procedure for conducting causal inference in LCA included: (1) PS estimation and evaluation of overlap, (2) calculation of inverse probability-of-treatment weighting (IPTW), (3) PS matching, (4) evaluating balance of confounding variables between exposure groups, and (5) conducting LCA using the weighted or matched data set.

**Results:**

Based on the results of IPTW which compared the low, medium and high risk classes of metabolic syndrome (compared to a class without metabolic syndrome), no association was found between smoking and the metabolic syndrome latent classes. PS matching which compared low and moderate risk classes compared to class without metabolic syndrome, showed that smoking increases the probability of being in the low-risk class of metabolic syndrome (OR: 2.19; 95% CI: 1.32, 3.63). In the unadjusted analysis, smoking increased the chances of being in the low-risk (OR: 1.45; 95% CI: 1.01, 2.08) and moderate-risk (OR: 1.68; 95% CI: 1.18, 2.40) classes of metabolic syndrome compared to the class without metabolic syndrome.

**Conclusions:**

Based on the results, the causal effect of smoking on latent hazard classes of metabolic syndrome can be different based on the type of PS method. In adjusted analysis, no relationship was observed between smoking and moderate-risk and high-risk classes of metabolic syndrome.

## Background

The American Heart Association and the National Heart, Lung, and Blood Institute have considered metabolic syndrome as presence of three or more metabolic syndrome components, including Increased waist circumference (abdominal obesity), hypertriglyceridemia, low HDL cholesterol, impaired fasting blood sugar, and hypertension [1, 2]. The metabolic syndrome prevalence is increasing worldwide and its prevalence varies in different parts of the world depending on environmental factors, sex, age, race and ethnicity [3, 4]. In Asian countries, the prevalence of this syndrome is between 10 and 20% [5–7]. The metabolic syndrome prevalence in Iranian youth is dramatically high, ranging from 4.8 to 24.5%. Interestingly, its prevalence in the elderly was significantly higher than in the young and 49.5% reported [8]. The risk of death, stroke and heart attack in people with metabolic syndrome is 2 to 3 times higher than healthy people. Also, metabolic syndrome increases the risk of diseases such as diabetes, cardiovascular disease, fatty liver, asthma, ovarian cysts and a number of cancers [9, 10].

Although metabolic syndrome is traditionally recognized as an ‘all or one’ condition, it is unknown whether this definition is accurate and to date has not been validated [11]. A proposed method for classifying metabolic syndrome is latent class analysis (LCA). LCA is a model that shows that there is a latent classification variable that divides population into latent classes [12]. Latent classes are created to show unobserved heterogeneity among individuals according to observed variables [9, 13]. Metabolic syndrome does not have a standard diagnostic test and the use of LCA can help to identify more [11].

Smoking is one of the major causes of mortality and disease in the world [14] and is responsible for about 7.2 million deaths per year. Moreover, smoking is one of the modifiable risk factors for non-communicable diseases such as cardiovascular disease and type 2 diabetes [15].

In previous studies, regression methods have often been used to investigate the relationship between smoking and metabolic syndrome and adjust for confounding variables [15–17]. An alternative approach for confounding adjustment is exposure modeling with propensity score (PS) methods. PS which is the conditional probability of exposure, given the set of measured confounders [18, 19] can be used in different procedures for balance of confounding variables among exposure groups, including matching, stratification, inverse probability-of-treatment weighting (IPTW), and use of the PS as a covariate [20–34, 83].

Although regression models are widely used in practice, PS methods are preferred for inferring causality for the following reasons: First, it is easier to determine whether the exposure models are adequately specified in terms of yielding covariate-balancing propensity scores using standardized differences. Second, these methods effectively emulate a randomized experiment without any reference to the outcome. Third, the overlap in the distribution of confounders can be explicitly assessed between two exposure groups [20].

Any analysis of observational data, including the effect of an exposure on latent class members, is subject to confounding and here we apply an approach to causal inference in LCA using PS methods. Therefore, the aim of this study was to investigate the causal effect of smoking on the latent hazard classes of metabolic syndrome using latent class causal analysis.

## Methods

### Participants

In this study, we used data from the Tehran Lipid and Glucose Cohort Study (TLGS), designed to investigate risk factors of non-communicable diseases. TLGS is an ongoing study that started in 1998 and performed in several phases. The current study was based on a sample of 4857 participants. We used the third phase data (2005–2008) as the baseline, and participants aged over 20 with complete information on the variables including age, gender, physical activity, marital status, education, job and smoking status were selected. In the third phase, people with metabolic syndrome criteria were excluded, that is, people who had 3 or more metabolic syndrome components. We measured the metabolic syndrome components to obtain latent classes of metabolic syndrome in the fifth phase data. (2011–2014). The TLGS main project has been approved by the IRB of the Iranian National Scientific Research Council and the Research Institute for Endocrine Sciences, Shahid Beheshti University of Medical Sciences, under the Helsinki Declaration and an informed consent form was obtained from all participants [35, 36]. The ethics committee of the School of Health, Tehran University of Medical Sciences (code of IR.TUMS.SPH.REC.1398.032) has approved this project.

.

### Measures

#### Exposure and outcome

The exposure variable was smoking measured by asking the question: “is person smoking daily?”. The outcome variable was metabolic syndrome. Components measuring metabolic syndrome, included abdominal obesity (waist circumference for men and women ≥ 95 cm), low HDL cholesterol (< 40 mg/dl in men or < 50 mg dl in women), hypertriglyceridemia (TG ≥ 150 mg/dl), hypertension (systolic blood pressure ≥ 130 mmHg or diastolic blood pressure ≥ 85 mmHg) and impaired blood glucose (fasting blood glucose ≥ 100 mg/dl) [37–39].

#### Confounders

**Fig. 1:**
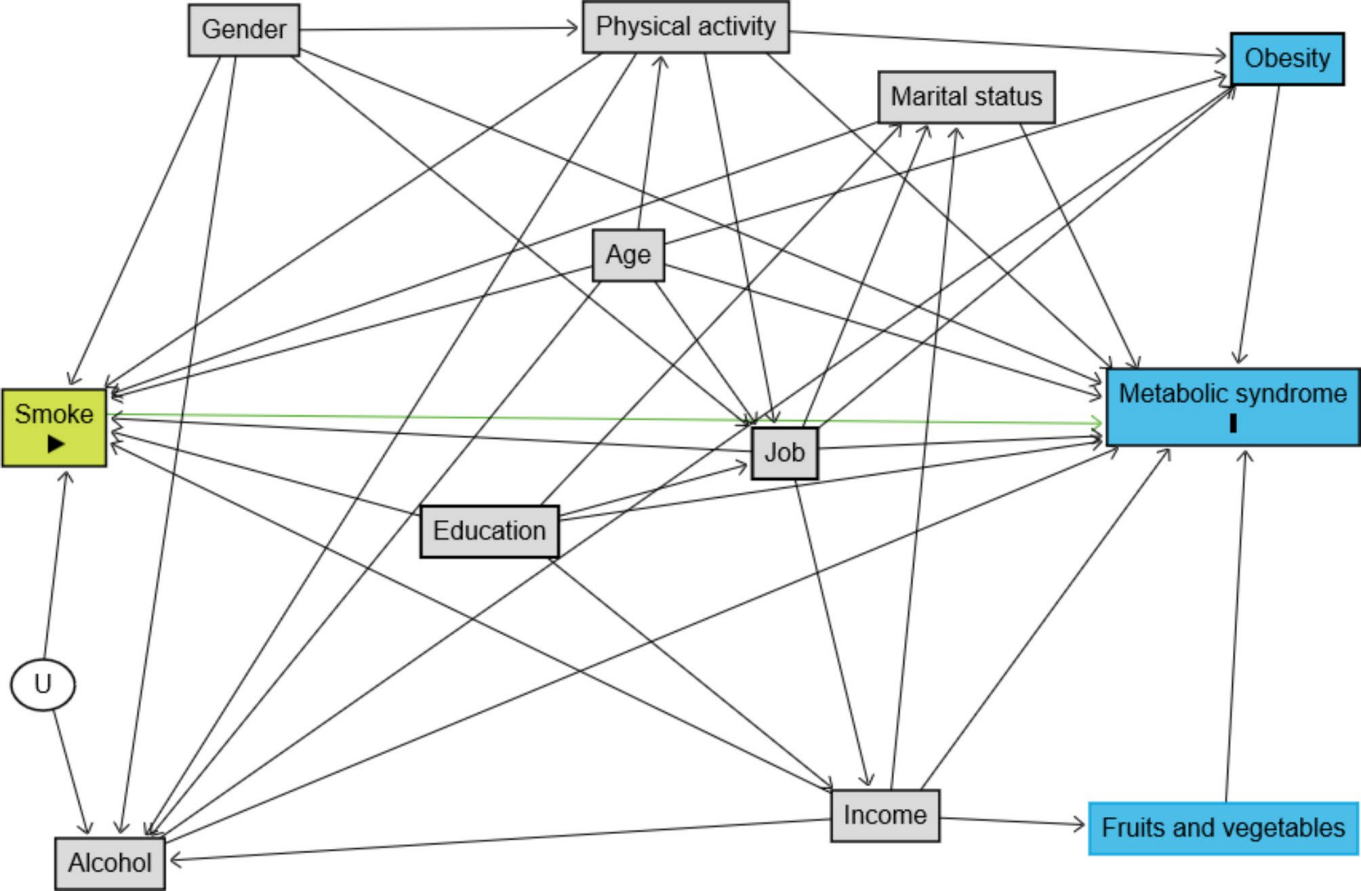
Causal diagram for the effect of smoking on MS

A causal-directed acyclic graph (cDAG) [40–48, 82] for the study population was created using the DAGitty package [49] (Fig. 1). The diagram shows the causal relationships between exposure, outcome and covariates which was drawn based on the literature review. The minimally sufficient set for confounding adjustment, derived based on Pearl’s back-door criterion [50], included gender, age, physical activity, marital status, education, and job as well as the unmeasured variables income and alcohol. Fractional polynomials were used to identify any nonlinear association between age and exposure (smoking) in the PS model [51–55].

### Statistical methods

***Steps of causal inference in LCA using PS methods***

#### Step 1: PS estimation and evaluation of overlap

PS, the probability of exposure conditional on confounders [19], was estimated through logistic regression, with smoking as the response variable and confounders as predictors. We evaluated $\widehat{PS}$ overlap in the exposed and unexposed groups using a histogram. The correlations between the predictors were assessed with the correlation matrix. The highest correlation was less than 0.3, so collinearity in the exposure model was not present.

#### Step 2: calculate IPTW and PS matching

***Inverse probability-of-treatment weighting (IPTW)***

IPTW was used to adjust for the minimally sufficient set of confounders. The rationale for weighting in IPTW is that over-represented persons (people with a high probability of exposure to cigarettes) take a low weight and under-represented persons (people with a low probability of exposure to cigarettes) take a high weight. Average treatment effect (ATE) in the whole population was estimated with weights equal to $\frac{1}{\widehat{PS}}$ for the smokers and $\frac{1}{(1-\widehat{PS})}$for the non-smokers. The rationale behind the IPTW is that it produces a pseudo-population in which confounders do not predict the exposure anymore, and the causal effect of interest in the pseudo-population is the same as that in the population [56–58].

#### PS matching

A PS-matched dataset was created by matching, without replacement, one unexposed person to one exposed based on the nearest value of $\widehat{PS}$ (± 0.05) [57]. Of note, a caliper width of 0.2 of the standard deviation of the logit of the PS [59] was deemed to be 0.25 which was considered too large for matching and thus was not included. The PS matching was performed using the R package Match It [60].

#### Step 3: evaluating balance of confounding variables between exposure groups

PS is a balancing score, so in persons with the same PS, the distribution of confounders should be similar between the exposed and unexposed. The correct specification of the PS model can be assessed based on the balance of measured confounders between exposure groups. The balance was evaluated in the matched sample for PS matching, and in the weighted sample for the IPTW. The standardized difference was used to compare the mean and proportion of continuous and binary confounders between the exposed and unexposed, respectively. The standardized difference for continuous confounders is defined as

$$d=\frac{({\stackrel{-}{x}}_{exposed}-{\stackrel{-}{x}}_{unexposed})}{\sqrt{\frac{{s}_{exposed}^{2}+{s}_{unexposed}^{2}}{2}}}$$

where ${\stackrel{-}{x}}_{exposed}$ and ${\stackrel{-}{x}}_{unexposed}$ are the mean estimates and ${s}_{exposed}^{2}$ and ${s}_{unexposed}^{2}$ are variance estimates in the exposed and unexposed, respectively.

The standardized difference for binary confounders is defined as

$$d=\frac{{\widehat{p}}_{exposed}-{\widehat{p}}_{unexposed}}{\sqrt{\frac{{\widehat{p}}_{exposed}\left(1-{\widehat{p}}_{exposed}\right)+{\widehat{p}}_{unexposed}(1-{\widehat{p}}_{unexposed})}{2}}}$$

where ${\widehat{p}}_{exposed}$ and ${\widehat{p}}_{unexposed}$ are the proportion estimates of the binary confounders in the exposed and unexposed, respectively.

Although there is no consensus on the cutpoint of the standardized difference for defining an important imbalance, a standardized difference of less than 0.1 was considered as an unimportant difference in mean or proportion of confounders between exposure groups [20].

#### Step 4: Conduct LCA using the weighted or matched data set

LCA is a latent variable model that classifies homogeneous individuals. LCA is used to find groups in classified data, which are called latent classes. LCA has two parameters, class prevalence and item-response probability. The probability of membership in each latent class is called class prevalence. Item-response probability is the conditional probability of “yes” response to metabolic syndrome components. The probability of “no” response can be calculated by subtracting item-response probabilities from 1. These probabilities constitute the basis for interpreting and naming latent classes: class in which all the metabolic syndrome components have a probability less than 0.5 as without metabolic syndrome, one component has a probability higher than 0.5 as low risk, two of components have a probability higher than 0.5 as moderate risk, and three or more components have a probability higher than 0.5 are considered high risk. To conduct LCA, five observed dichotomous variables (metabolic syndrome components) were used to classify metabolic syndrome as a latent variable.

We conducted the LCA model in three types of data, original data, weighted data using IPTW, and matched data using PS matching. To select the best model, we compared LCA models 1 to 6 classes. Akaike’s Information Criterion (AIC) [61], Bayesian Information Criterion (BIC) [62], Consistent Akaike’s Information Criterion (CAIC) [61], and Adjusted Bayesian Information Criterion (ABIC) [63] were used to select the best model. Lower values of these indices indicate better model fit. Next, in each type of data, we examined the relationship between smoking and the latent classes of metabolic syndrome through multinomial logistic regression model and estimated the odds ratio (OR) with 95% confidence interval (CI) [64, 65]. The 95% CIs for the IPTW estimates were derived using robust standard errors [66]. The 95% CIs for the PS matching was obtained based on nonparametric bootstrapping by 1000 repetitions with 2.5th and 97.5th percentiles as 95% confidence limits [67].

#### Software

R software was used to perform IPTW and PS matching analyses and calculate the standardized differences for confounders. The PS matching was performed using R package Match It [60]. The R package tableone was used for calculating standardized differences for IPTW and PS matching [68]. SAS package PROC LCA was used to obtain LCA [69].

## Results

Of the 4857 participants included in this study, 2959 (60.9%) were female, and the mean (standard deviation) of age of participants was 39.10 (13.48) years, ranging from 20 to 90. Moreover, there were 512 (10.5%) cigarette smokers at baseline. In the PS matching, 508 unexposed subjects were matched to 508 exposed subjects. The mean (SD) of inverse probability-of-treatment weights for ATE estimate was 2.02 (5.57). The baseline characteristics of participants based on smoking have been shown in Table 1.

**Table 1:** Baseline characteristics of the study participants

Characteristic	Smokers, n (%)	Non-smokers, n (%)
Gender (female)	74(14.5)	2885 (66.4)
Age, years, mean (SD)	40.89 (12.67)	38.89 (13.56)
Marital status		
single	102 (19.9)	918 (21.1)
married	391 (76.4)	3233 (74.4)
divorced	13 (2.5)	56 (1.3)
widowed	6 (1.2)	138 (3.2)
Educational certificate		
elementary	57 (11.1)	592 (13.6)
secondary school	98 (19.1)	641 (14.8)
high school	264 (51.6)	2067 (47.6)
associate degree	31 (6.1)	284 (6.5)
BSc	56 (10.9)	653 (15.0)
MSc or higher degrees	6 (1.2)	108 (2.5)
Occupational status		
employed	378 (73.8)	1606 (37.0)
student	11 (2.1)	291 (6.7)
housewife	51 (10.0)	2039 (46.9)
no work with income	46 (9.0)	325 (7.5)
others	26 (5.1)	84 (1.9)
Physical activity (yes)	310 (60.5)	2812 (64.7)

**Fig. 2:**
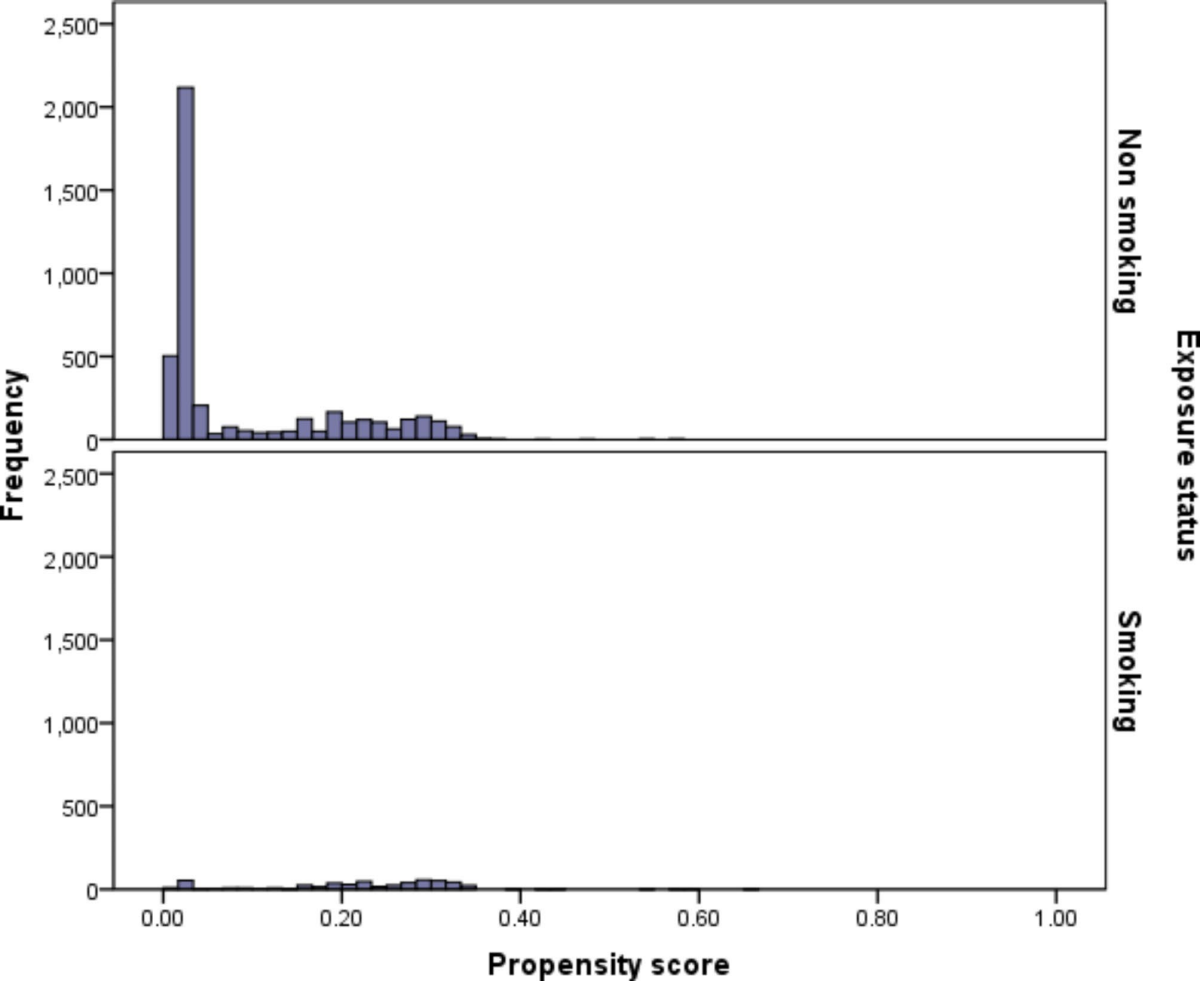
Histogram diagram of estimated propensity scores for the exposed and unexposed groups

Figure 2 shows the $\widehat{PS}$ overlap in the exposed and unexposed groups using a histogram. Based on the figure, the distribution of $\widehat{PS}$ in the smoker and non-smokers groups shows that there is sufficient overlap between two groups.

Table 2 represents the standardized differences for confounders in original, weighted and matched data. In the original data, eight variables had standardized differences above 0.1, but in both weighted and matched data, all variables had standardized differences less than 0.1, indicating that a sufficient balance on the confounders was established between exposure groups.

**Table 2:** Standardized differences before (unadjusted) and after (adjusted) applying IPTW and use of PS matching

Confounder	Original data	Weighted data using IPTW	Matched data using PS matching
Gender (male)	1.248	0.020	0.011
Age (year)	0.184	0.018	0.016
Marital status			
single	0.030	0.015	0.019
divorced	0.091	0.019	0.015
widowed	0.138	0.030	0.077
Educational certificate			
elementary	0.076	0.087	0.006
Secondary school	0.117	0.015	0.005
associate degree	0.020	0.007	0.024
BSc	0.122	0.006	0.006
MSc or higher degrees	0.098	0.062	0.017
Occupational status			
student	0.223	0.002	0.026
housewife	0.898	0.019	0.020
no work with income	0.055	0.045	0.007
others	0.171	0.013	0.038
Physical activity	0.086	0.019	0.024

Abbreviations: IPTW, inverse probability-of-treatment weighting; PS, propensity score

Table 3 compared LCA models 1 to 6 classes in the original data, weighted data using IPTW, and matched data to select the best model. Based on this table, in the weighted data using IPTW, the four-class model had the lowest values of BIC, CAIC, and ABIC, and the five-class model had the lowest value of AIC. Based on the lower values of BIC, CAIC and ABIC indicators, we preferred the four-class model for the weighted data. In the matched data, the three-class model had the lowest values of AIC, BIC, CAIC and ABIC. Therefore, based on the lower values of these indicators, the three-class model was preferred. Based on the original data, the three-class model had the lowest values of BIC, CAIC and ABIC, and the four-class model had the lowest value of AIC. Based on lower values of BIC, CAIC and ABIC indicators, the three-class model was preferred.

**Table 3:** Summary of information for selecting number of latent classes for metabolic syndrome

Method	No. of Classes	AIC	BIC	CAIC	ABIC	N
ATE: Weighting	1	889	921.4	926.4	905.5	4857
	2	371.3	442.7	453.7	407.7	
	3	175.9	286.2	303.2	232.2	
	4	99.2	248.4	271.4	175.3	
	5	78.6	266.7	295.7	176.6	
	6	78.7	305.8	340.8	194.6	
ATT: Matching	1	141.9	166.5	171.5	150.7	1016
	2	87.2	141.4	152.4	106.4	
	3	46.4	130.1	147.1	76.1	
	4	49.1	162.4	185.4	89.3	
	5	59.5	202.3	231.3	110.2	
	6	70.8	243.1	278.1	132	
Original data	1	838.1	870.6	875.6	854.7	4857
	2	294.7	366	377	331.1	
	3	75	185.3	202.3	131.3	
	4	50.5	199.8	222.8	136.7	
	5	59	247.2	276.2	155.1	
	6	70.7	297.8	332.8	186.6	

Abbreviations: AIC, Akaike’s Information Criterion; BIC, Bayesian Information Criterion; CAIC, Consistent

Akaike’s Information Criterion; aBIC, Adjusted Bayesian Information Criterion; ATE, Average treatment effect;

ATT, Average treatment effect in the treated

Table 4 shows the item-response probabilities for the four-class, three-class and three-class metabolic syndrome models in the weighted data using IPTW, matched data, and original data. Probability higher than 0.5 was considered as high probability. Based on this table, the class without metabolic syndrome shows people who had a low probability (less than 0.5) of metabolic syndrome components: in the weighted, matched, and original data, they comprised of 22%, 30%, and 57% of the population, respectively. Based on Table 4 in the weighted data using IPTW, the low-risk class comprised of 31% of the population and included people at high-risk of abdominal obesity. The moderate-risk class comprised of 39% of the population and also included people who were at a higher risk for hypertriglyceridemia and low HDL cholesterol levels. The high-risk class comprised of 8% of the population and included people who are at high-risk for all metabolic syndrome components except hypertension. In the matched data, the low-risk class comprised of 36% of the population and included people at high-risk for low HDL cholesterol. The moderate-risk class was comprised of 34% of the population and included people at high-risk of abdominal obesity and hypertriglyceridemia. In the original data, the low-risk class comprised of 27% of the population and included people who are at high-risk of abdominal obesity. The moderate-risk class comprised of 16% of the population and included people at high-risk for hypertriglyceridemia and low HDL cholesterol.

**Table 4:** Item-response probabilities for the four-class, three-class and three-class metabolic syndrome models in IPTW-weighted data, PS-matched data and original data

		ATE: Weighting				ATT: Matching				Original data		
		Class 1 (No metabolic syndrome)	Class 2 (Low risk)	Class 3 (moderate risk)	Class 4 (High risk)	Class 1 (No metabolic syndrome)	Class 2 (Low risk)	Class 3 (moderate risk)		Class 1 (No metabolic syndrome)	Class 2 (Low risk)	Class 3 (moderate risk)
		(22%)	(31%)	(39%)	(8%)	(30%)	(36%)		(34%)	(57%)	(27%)	(16%)
	Response											
abdominal obesity	Yes	0.034	**0.550**	0.254	**0.780**	0.300	0.260		**0.675**	0.179	**0.716**	0.406
impaired blood glucose	Yes	0.093	0.319	0.153	**0.536**	0.185	0.181		0.440	0.132	0.386	0.279
Hyper triglyceride	Yes	0.002	0.215	**0.591**	**0.987**	0.000	0.494		**0.509**	0.080	0.352	**0.943**
Hypertension	Yes	0.003	0.413	0.073	0.419	0.183	0.062		0.479	0.107	0.479	0.207
Low HDL cholesterol	Yes	0.075	0.172	**0.616**	**0.691**	0.000	**0.574**		0.314	0.260	0.244	**0.790**

Note. Item-response probabilities > 0.50 appear in bold to facilitate interpretation

Abbreviations: ATE, Average treatment effect; ATT, Average treatment effect in the treated

Table 5 shows the causal effect of smoking on metabolic syndrome latent classes in the weighted data using IPTW and matched data. Also, this table shows the effect of smoking on the metabolic syndrome latent classes in the original data. In the matched data, smoking increased the chances of being in the low-risk class of metabolic syndrome (OR: 2.19; 95% CI: 1.32, 3.63) compared to the class without metabolic syndrome. Based on the matched data, 95% CI was compatible with both increase and decrease chance of being in moderate-risk class of metabolic syndrome. The same pattern of inconclusive CIs was seen for being in the low, moderate, and high risk class in the weighted analysis.

**Table 5:** Odds ratios and confidence intervals for the relationship between smoking and latent classes of metabolic syndrome before (unadjusted) and after (adjusted) applying IPTW and use of PS matching

	Classes	Odds Ratio	95% Confidence interval
ATE: Weighting	No metabolic syndrome	reference	reference
	Low risk	0.487	0.185, 1.28
	moderate risk	1.52	0.463, 4.98
	High risk	0.918	0.266, 3.16
ATT: Matching	No metabolic syndrome	reference	reference
	Low risk	2.19	1.32, 3.63
	moderate risk	0.842	0.487, 1.45
Original data	No metabolic syndrome	reference	reference
	Low risk	1.45	1.01, 2.08
	moderate risk	1.68	1.18, 2.40

## Discussion

In this cohort study, we investigated the causal effect of smoking on the latent hazard classes of metabolic syndrome by integrating causal inference methods in the LCA. Integration of PS methods in LCA provides a better understanding of the causal mechanism of behavior or characteristics that are not directly measurable and allows researcher to easily control for many confounders simultaneously [56]. The most important advantage of using the causal inference approach to estimate each effect is that it allows researcher needs to ask a specific causal question. Average treatment effect (ATE) and average treatment effect in the treated (ATT) expresses different questions that differ in terms of the population to which we generalize the results. We first estimated ATE by IPTW, which ask” if everyone in the community smoked, what difference is expected in the pattern of metabolic syndrome compared to those who had never smoked”. We subsequently estimated ATT by PS matching, which asks “among people who smoked, assuming they all did,, what difference is expected in the pattern of metabolic syndrome compared to those who had never smoked” none of them having smoked? We believe that ATT provides us with a more realistic feeling. In fact, to answer this question, we are comparing smokers in terms of actual behavior of smoking with their expected behavior if they do not smoke. Thinking about the expected effects of smoking in the whole population does not seem logical, because in practice, many people in a population do not smoke (since different people in society have different behavioral characteristics). The results by PS matching, which considers exposed individuals, appear to be more plausible than the results of the IPTW, which measures the whole population.

In causal LCA, we first selected the model and obtained the relationship between smoking and metabolic syndrome latent classes by comparing the low, medium and high risk classes of metabolic syndrome with class without metabolic syndrome through polynomial regression. Based on the results of unadjusted model, we considered the three-class model, which included people without metabolic syndrome (57%), people with low risk of metabolic syndrome (26%) and people with moderate risk of metabolic syndrome (15%). Based on this model, smoking increased the chances of being in the low-risk and moderate-risk classes of metabolic syndrome compared to the class without metabolic syndrome. The purpose of adjustment and inclusion of IPTW and PS matching in the model is to analyze by re-weighing everyone based on IPTW or matching and removing confounders similar to a randomized controlled trial. Based on IPTW, no association was found between smoking and the metabolic syndrome latent classes.

In a study from the Czech Republic on 805 people in the age group of 18 to 65 years, the prevalence of metabolic syndrome did not differ between smokers and non-smokers [70]. In another study by Ishizaka et al. in Japan on 3687 people, smoking was not a predictor of metabolic syndrome and no relationship was found between these two variables [71]. In a study by Santos et al. in Porto, Portugal on 2165 people in the age group of 18 to 92 years, the prevalence of metabolic syndrome was not different in smokers and non-smokers [72]. Based on PS matching, smoking increased the chances of being in the low-risk class of metabolic syndrome compared to the class without metabolic syndrome. Previous studies have shown an association between smoking and metabolic syndrome, for example, in a multinational study with different ethnicities, non-Hispanic white, African-American, Hispanic, and American-Chinese, conducted in six U.S. regions, smoking increased the chances of developing metabolic syndrome compared to those who did not smoke [73]. In addition, Slagter et al. conducted a study in the Netherlands with 59,467 people, observed a higher prevalence of metabolic syndrome in smokers [17]. Sun et al. Conducted a meta-analysis of several cohort studies in different parts of Asia, Europe and North America, they found that smoking increases the risk of metabolic syndrome [74]. Also, an increased risk of metabolic syndrome associated with smoking, was observed in results of other studies in other parts of the world [1, 75–77]. The effects of smoking on the cardiovascular system can be caused by an increase in nicotine receptors. Nicotine receptor activation can secrete neurotransmitters and hormones such as vasopressin, growth hormone, dopamine, serotonin and glutamate in the central nervous system, acetylcholine in the peripheral nervous system, and catecholamine and cortisol from the adrenal glands. All these molecules affect metabolism [78]. Also, studies show that smoking causes inflammation, which predisposes to metabolic syndrome. Smoking increases the production of procytokines, decreases the level of anti-inflammatory cytokines, and increases the pathological level of inflammatory-sensitive proteins such as Alpha 1-antitrypsin and fibrinogen [79].

This study has some limitations. First, the validity of the causal analyses using this study relies on no unmeasured confounding. However, some confounders such as alcohol consumption and income were not available. Although we did not have income data, job and education variables were included in the model as suitable proxies for income. Alcohol is expected to have a positive relationship with smoking and also outcome: had adjusted for it, the effect estimate would have been even weaker than the current estimate. Second, there might have been some measurement bias as smoking was dichotomized and self-reported so subject to recall and under-reporting biases [80, 81]. Third, some adjusted confounders like physical activity might have had measurement error leading to residual confounding. Fourth, the exclusion of the subjects with missing data on baseline confounders might be subject to selection bias. However, only 10% of the participants were excluded for this reason with the mean age of 41 years and 57% female, which is somewhat similar to the people included in the study.

## Conclusion

In summary, the results of this study showed that in unadjusted analyses, there were association between smoking and the chances of being in the low-risk and moderate-risk classes of metabolic syndrome compared to the class without metabolic syndrome, but after adjustment with IPTW, no strong evidence of an association between smoking and metabolic syndrome latent classes was observed Based on PS matching, smoking increased the chances of being in the low-risk class of metabolic syndrome compared to the class without metabolic syndrome. The differences in results can be explained by no confounding adjustment in the unadjusted analysis as well as different effect targets for the IPTW and PS matched adjusted analyses.

## Data Availability

Data are available from the authors upon reasonable request to the corresponding authors.

## Abbreviations

LCA: Latent class analysis
IPTW: Inverse probability of treatment weighting
PS: Propensity score
OR: Odds ratio
CI: Confidence interval
AIC: Akaike information criterion
BIC: Bayesian information criterion
CAIC: Consistent Akaike’s Information Criterion
ABIC: Adjusted Bayesian Information Criterion
ATE: Average treatment effect
